# Frequency-Dependent Sonochemical Processing of Silicon Surfaces in Tetrahydrofuran Studied by Surface Photovoltage Transients

**DOI:** 10.3390/molecules26123756

**Published:** 2021-06-20

**Authors:** Artem Podolian, Andriy Nadtochiy, Oleg Korotchenkov, Viktor Schlosser

**Affiliations:** 1 Faculty of Physics, Taras Shevchenko National University of Kyiv, 01601 Kyiv, Ukraine; summer.podolian@gmail.com (A.P.); namobem@gmail.com (A.N.); olegk@univ.kiev.ua (O.K.); 2 Department of Electronic Properties of Materials, Faculty of Physics, University of Vienna, A-1090 Wien, Austria

**Keywords:** power ultrasound, sonochemistry, ultrasonic frequency, silicon, tetrahydrofuran, surface photovoltage

## Abstract

The field of chemical and physical transformations induced by ultrasonic waves has shown steady progress during the past decades. There is a solid core of established results and some topics that are not thoroughly developed. The effect of varying ultrasonic frequency is among the most beneficial issues that require advances. In this work, the effect of sonication of Si wafers in tetrahydrofuran on the photovoltage performance was studied, with the specific goal of studying the influence of the varying frequency. The applied ultrasonic transducer design approach enables the construction of the transducer operating at about 400 kHz with a sufficient sonochemical efficiency. The measurements of the surface photovoltage (SPV) transients were performed on p-type Cz-Si(111) wafers. Sonication was done in tetrahydrofuran, methanol, and in their 3:1 mixture. When using tetrahydrofuran, the enhanced SPV signal (up to ≈80%) was observed due to increasing sonication frequency to 400 kHz. In turn, the signal was decreased down to ≈75% of the initial value when the frequency is lowered to 28 kHz. The addition of methanol suppressed this significant difference. It was implied that different decay processes with hydrogen decomposed from tetrahydrofuran could be attempted to explain the mechanism behind the observed frequency-dependent behavior.

## 1. Introduction

It is generally accepted that the chemical effect of ultrasound in a chemical mixture results primarily from cavitation hot spots although it has been made clear that chemical transformations can also occur in ultrasonic fields without cavitation [1]. Due to locally achieved extreme temperature and pressure conditions, an unusual chemical environment is often achieved using sonochemical method [2]. A growing body of research data shows that sonochemical synthesis of different materials, particularly in nanophases, is useful and promising [3,4,5]. Using the ultrasonic technique, excellent emulsification and dispersion can be achieved [6,7]. Acoustic cavitation is also useful for efficient surface cleaning [8]. Sonochemical processing of semiconductor surfaces has been studied rather more recently [9,10,11,12,13,14,15]. Near-surface distribution of excess charge carriers can have very different properties in semiconductors and semiconductor nanostructures, depending on morphology, temperature, and surface properties such as dangling bond defects of different configurations, the presence of a suboxide interlayer, and bonds between adsorbates and surface atoms, etc. The sonochemical processing technique can therefore provide a unique tool to modify the electronic properties following the response to the bubble collapse and breaking the chemical bonds on the surface in a variety of materials. This is particularly helpful for the processing of silicon whose surface electronic properties can very intriguingly be influenced by the chemical preparation [16].

The operating frequency range of sonochemical apparatus is typically up to dozens of kHz. A key assumption of choosing the frequency is that the size of the cavitation bubble is inversely related to the ultrasonic frequency. Therefore, because the bubble size drops with increasing the ultrasonic frequency and the bubble implosions become less violent, the energy released by each imploding cavitation bubble decreases with the ultrasonic frequency. However, the number of the imploding events increases due to the increased number of sound waves passing through the liquid at a higher frequency [17].

The computed bubble radius ranges from 0.1 to 100 μm at 20 kHz and from 0.1 to 3 μm at 1 MHz [18]. Above ≈1 MHz, the bubble disintegrates into smaller bubbles in a few acoustic cycles, while in the dozens and hundreds kHz frequency range, the bubble shape remains stable. The amount of water vapor in the collapsing bubble decreases with increasing the frequency. This behavior affects the multibubble sonoluminescence response, so that it originates mainly from the plasma emission at about 1 MHz while the involvement of OH radicals in the emissions was observed at 20 kHz [18].

Ultrasonic treatment at tuned frequencies has gained a great deal of interest in bio-medical research, e.g., being used to inactivate bacteria cells through cavitation [19]. Interestingly relevant to the scope of this work, the inactivation effects, which are different at a low frequency of 20 kHz and higher frequencies of 580 and 1146 kHz, arise from the collapse of acoustic cavitation bubbles that generates both physical and chemical effects, as was reported previously [20,21]. Shock waves and shear forces produced by the bubble collapse and acoustic streaming are obviously among the physical effects. Free radicals, HO^−^, O_2_^−^, and H^+^, as well as other kinds of oxidants originate from the decomposition of water vapor within the collapsing bubble. If volatile solutes are dissolved in the sonicated mixture, they enter the bubbles by evaporation and are consequently dissociated as well. The resulting chemical products can then diffuse outside the bubble, dissolving in the surrounding liquid and producing a variety of chemical reactions.

However, ultrasonic frequency is only one of the most important characteristic quantities describing the chemical effect of ultrasound [22]. Thus, varying sonochemical activity with stirring is a very important feature of the sonochemical process [23]. Reactions occurring with a high reaction rate are randomly distributed inside a reaction vessel, so that the stirring time would be shorter than the reaction time in order to complete the reaction in sonochemical instruments [24].

Moreover, the frequency of irrigation is crucial for chemical processes utilizing sonochemical devices [22]. In particular, passive ultrasonic irrigation implies the acoustic energy transmission aiming to activate the irrigant during the root canal treatment in dentistry [25]. Most generally, the pressure propagation through a liquid medium produces negative acoustic pressure, thus overcoming the tensile strength in the liquid medium and forming cavitation bubbles. In endodontics, this pressure change arises in the confined root canal [26].

Here, silicon wafers have been sonicated in tetrahydrofuran solutions. The surface properties were investigated by surface photovoltage (SPV) measurements. The results are reported and discussed. Tetrahydrofuran was used as a hydrogen promoter which, in turn, can induce the reconstruction of active centers at the Si surface that were associated with an appreciable modification of the charge carriers trapping and surface recombination dynamics. The influence of the ultrasonic frequency in the range from dozens to hundreds of kHz on the kinetic processes of photogenerated electrons and holes at silicon surfaces was demonstrated.

## 2. Materials and Methods

Different ultrasonic experimental arrangements have been utilized to achieve sonochemical treatments [3]. Most laboratory setups use either some ultrasonic bath or commercial ultrasonic horn systems. In this work, a low frequency sonication at about 28 kHz was performed in a standing wave ultrasonic bath using a Langevin transducer, as described elsewhere [27]. In developing a higher-frequency setup, it is important to keep in mind that the time for bubble creation may be longer in this case. Thus, at 20 kHz, the rarefaction cycle is 25 μs with peak pressure amplitude achieved in 12.5 μs, whereas the appropriate cycle is only 0.025 μs at the frequency of 20 MHz [28]. Consequently, one can conclude that the bubble production likelihood drops with the frequency, so that greater acoustic powers should be used in order to overcome the cohesive forces in a chemical mixture over shorter periods of the rarefaction cycles. For example, increasing the operating frequency from 10 to 400 kHz requires an order of magnitude enhancement in ultrasonic power to maintain cavitation in water [29]. The solid horns are most commonly employed in sonochemistry, material forming, processing, and medical applications to deliver high-intensity ultrasonic energy. Various device designs and design formulas have been widely considered [29,30]. An interesting multi-domain high-frequency LiNbO_3_ plate design has been proposed towards miniaturization of the resonator [31].

Here, a piezoceramic transducer operating at about 400 kHz was designed to maximize the acoustic power stored in the ultrasonic bath and thus to improve the energy efficiency of the sonochemical processing process.

### 2.1. Designing Ultrasonic Transducer

**Figure 1:**
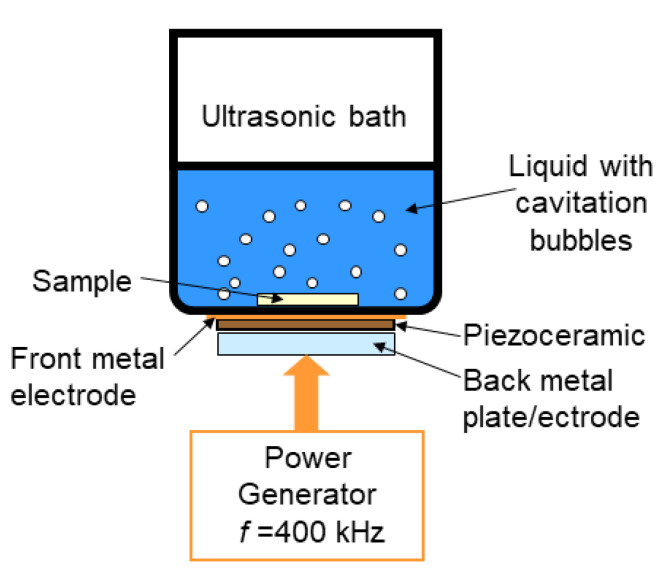
Schematics of the setup used for sonication at about 400 kHz.

A processed silicon wafer sample is placed at the bottom of a flask filled with a liquid reactant mixture, as shown in Figure 1. The piezoceramic transducer, placed between a front metal electrode and back metal plate, is externally glued to the bottom surface of the flask and generates an ultrasonic wave inside the mixture.

**Figure 2:**
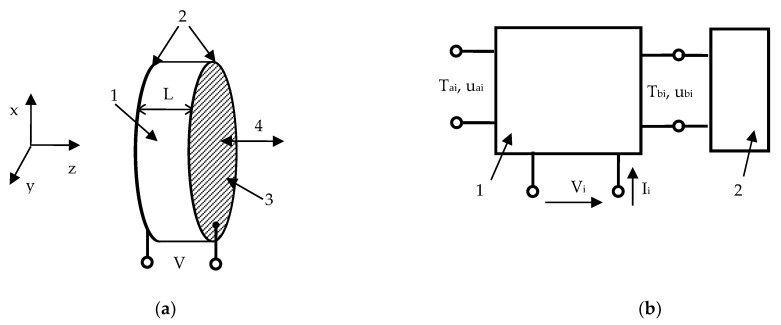
(**a**) Schematics of the longitudinal piezoelectric transducer: 1—piezoceramic disk, 2—metal electrode plates, 3—working surface of the transducer, displacement directions, V—applied rf voltage; (**b**) six-pole network model (1) for the transducer with an acoustic load (2).

We considered a piezoceramic disk with the thickness of L and poled in the z− direction, as shown in Figure 2a. The left and right sides of the plate were metalized. In the general case, the vibration of the piezoelectric body is described by the following equations [32]:

(1) $$\rho \frac{\partial^{2}u_{i}}{\partial t^{2}}=\frac{\partial T_{ij}}{\partial x_{j}},$$

(2) $$\frac{\partial D_{i}}{\partial x_{i}}=0,$$

(3) $$T_{ij}=c_{ijkl}^{E}\frac{\partial u_{k}}{\partial x_{l}}+e_{mij}\frac{\partial \varphi }{\partial x_{m}},$$

(4) $$D_{i}=e_{ikl}\frac{\partial u_{k}}{\partial x_{l}}-\varepsilon_{ij}^{S}\frac{\partial \varphi }{\partial x_{j}},$$

where $x_{j}$ is the coordinate (j= 1, 2, 3), $u_{i}$ is the displacement vector component (i=1, 2, 3), t is time, ρ is the mass density, $T_{ij}$ is the stress tensor, $D_{i}$ is the electric displacement vector, $c_{ijkl}^{E}$ are the elastic moduli of the medium under constant electric field, $e_{mij}$ are its piezoelectric coefficients, $\varepsilon_{ij}^{S}$ is the permittivity tensor under constant strain $S_{ik}=\frac{1}{2}\left(\frac{\partial u_{i}}{\partial x_{k}}+\frac{\partial u_{k}}{\partial x_{i}}\right)$, and repeated indices are summed.

Given that the thickness of the transducer was much smaller than the diameter of the disk, we could consider a one-dimensional (1D) vibration body. Assuming the wave is a harmonic plane wave traveling in the z− direction, the displacement components are defined by:

(5) $$u_{l}=u_{0l}e^{j\left(\omega t-kz\right)},$$

where j is the imaginary unit, k is the wave number, and ω is the angular frequency of the wave. Using complex wave numbers $k=k^{\prime }-jk^{\prime \prime }$ allowed us to take into account the losses in the transducer.

Substituting Equation (5) with l= 1, 2, 3 into Equations (1)–(4) provided a few solutions for one longitudinal and two transverse waves. In our particular case, only the longitudinal wave with the velocity of $V_{l}=\sqrt{c_{33}/\rho }$ was relevant to the transducer shown in Figure 2a. In this case, there would be two longitudinal waves counterpropagating in the z and −z directions. Omitting the exponential term $e^{j\omega t}$ and taking into account that only the $E_{3}\left(z\right)$ component of the electric field strength is not equal to zero, one equates:

(6) $$u_{1}=a_{1}e^{-jkz}+b_{1}e^{jkz},$$

(7) $$\varphi =-\int^{}E_{3}dz$$

The electrical current through the transducer is given by $I=j\omega AD_{3}$, with A being the cross-section of the transducer.

Dividing the transducer into a stack of n thin layers, the displacement in the *i*-th layer takes the form:

(8) $$u_{1}=a_{1}e^{-jk\left(z-z_{i}\right)}+b_{1}e^{jk\left(z-z_{i}\right)},$$

which results in the stress in the *i*-th layer:

(9) $$T_{i}=c_{i}jk_{i}\left(-a_{i}e^{-jk_{i}\left(z-z_{i}\right)}+b_{i}e^{jk_{i}\left(z-z_{i}\right)}\right)-e_{i}E_{3},$$

where $c_{i}=c_{33}^{E}+e_{33}^{2}/\varepsilon_{33}^{S}$ and $e_{i}=e_{33}$ for the *i*-th layer. The voltage drop across the *i*-th layer is then given by:

(10) $$V_{i}=-\int_{z_{i}}^{z_{i+1}}E_{3}dz=\frac{e_{i}}{\varepsilon_{i}}\left(a_{i}\left(e^{-jk_{i}L_{i}}-1\right)+b_{i}\left(e^{jk_{i}L_{i}}-1\right)\right)+j\frac{I_{i}L_{i}}{\omega A\varepsilon_{i}},$$

where $\varepsilon_{i}$ and $I_{i}$ are the permittivity and the current in the *i*-th layer, respectively, and $L_{i}=z_{i+1}-z_{i}$ is the thickness of the *i*-th layer.

One can then employ the impedance matrix approach to describe the transducer [32,33]. For this, the boundary conditions for the *i*-th layer take the form:

(11) $$u_{ai}={u_{i-1}|}_{z=z_{i}}={u_{i}|}_{z=z_{i}},u_{bi}={u_{i}|}_{z=z_{i+1}}={u_{i+1}|}_{z=z_{i+1}},\;T_{ai}={T_{i-1}|}_{z=z_{i}}={T_{i}|}_{z=z_{i}},T_{bi}={T_{i}|}_{z=z_{i+1}}={T_{i+1}|}_{z=z_{i+1}}$$

Using Equations (10) and (11), one writes the following system of three linear equations:

(12) $$\left[\begin{array}{c} T_{ai}\\ T_{bi}\\ V_{i} \end{array}\right]=\left[\begin{array}{ccc} -\frac{c_{i}k_{i}}{\tan k_{i}L_{i}} & \frac{c_{i}k_{i}}{\sin k_{i}L_{i}} & \frac{je_{i}}{\omega A\varepsilon_{i}}\\ -\frac{c_{i}k_{i}}{\sin k_{i}L_{i}} & \frac{c_{i}k_{i}}{\tan k_{i}L_{i}} & \frac{je_{i}}{\omega A\varepsilon_{i}}\\ -\frac{e_{i}}{\varepsilon_{i}} & \frac{e_{i}}{\varepsilon_{i}} & \frac{jL_{i}}{\omega A\varepsilon_{i}} \end{array}\right]\cdot \left[\begin{array}{c} u_{ai}\\ u_{bi}\\ I_{i} \end{array}\right]$$

where $e_{i}=e_{33}$ and $\varepsilon_{i}=\varepsilon_{33}^{S}$.

The square 3 × 3 matrix was a three-port impedance matrix. Since each of the three ports had two transmission lines, a six-pole network was formed, as shown in Figure 2b. The network had two acoustic ports, which are marked as $T_{ai},u_{ai}$ and $T_{bi},u_{bi}$ in Figure 2b, and one electrical port ($V_{i}$). In the case of non-piezoelectric vibrating disks, the electrical port was absent in the network, so one would consider only two acoustic ports shown in Figure 2b. Hence, a solid layer of finite thickness is a two-port element forming a four-pole network. With increasing layer thickness to infinity, one would attain a two-pole network or a one-port element, which acts as the absorber of ultrasonic waves. At the absorber edge, the amplitude of the forward wave traveling from infinity to the port was equal to zero such that the stress and displacement could be related through the acoustic impedance Z as $T_{b}=Z_{b}u_{b}$.

If the six-pole network p is acoustically loaded by one-port elements with the impedances $Z_{a}$ and $Z_{b}$ at the left- and right-hand sides, respectively, the resistance of the appropriately loaded transducer can be found as:

(13) $$R=p_{3,3}+\frac{p_{3,1}\left(Z_{b}p_{1,3}+p_{1,2}p_{2,3}-p_{1,3}p_{2,2}\right)+p_{3,2}\left(Z_{a}p_{1,2}+p_{1,3}p_{2,1}-p_{1,1}p_{2,3}\right)}{Z_{a}Z_{b}-Z_{a}p_{2,2}-Z_{b}p_{1,1}+p_{1,1}p_{2,2}-p_{1,2}p_{2,2}}\;$$

The four-pole network q connected to a one-port element with the impedance $Z_{b}$ at the right-hand side, as shown in Figure 2b, becomes a two-pole network with:

(14) $$Z_{a}=\frac{Z_{b}q_{1,1}-q_{1,1}q_{2,2}+q_{1,2}q_{2,1}}{Z_{b}-q_{2,2}}\;$$

Instead, the four-pole network q connected to a one-port element with the impedance $Z_{a}$ at the left-hand side has:

(15) $$Z_{b}=\frac{Z_{a}q_{2,2}-q_{1,1}q_{2,2}+q_{1,2}q_{2,1}}{Z_{a}-q_{1,1}}\;$$

**Figure 3:**
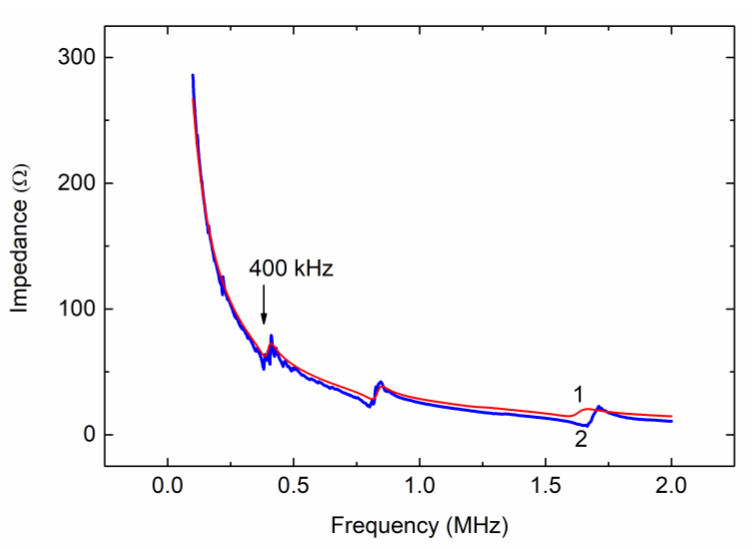
Calculated (1) and measured (2) impedance magnitude response for piezoelectric transducer shown in Figure 2a.

Therefore, using Equations (14) and (15), the transducer with any number of loading layers can be described by the two-pole network with the resistance given by Equation (13). Our computation result is shown by curve 1 in Figure 3.

To experimentally realize the ultrasonic reactor setup shown in Figure 1, which operates in the frequency range from about 300 to 500 kHz, we used a round-bottom glass flask with a diameter of about 40 mm and a bottom thickness of 3.1 mm. Front brass electrode was about 0.1 mm, whereas the back metal plate and electrode (see Figure 1) were round disks of 40 mm in diameter and 2 mm in thickness.

The measured frequency dependence of the impedance, which is shown by curve 2 in Figure 3, demonstrated an excellent agreement with the computed impedance response (curve 1). It was reliably identified that the resonance at about 400 kHz marked by arrow in Figure 3 was due to a coupled oscillator, which represented the lowest-vibration mode in an acoustic system composed of the piezoceramic, backing, front metal layers, and water load (see Figure 1). Scanning the driving frequency, we observed intense acoustic cavitation in a liquid poured into the flask just at the frequency of about 400 kHz, thus verifying the essential correctness of the transducer fabrication and quantitation approach.

To compare sonochemical efficiency at the frequencies of about 28 and 400 kHz used here, standard calorimetric measurements [34] were performed. For this purpose, 20 mL of water was sonicated at 28 and 400 kHz. In both cases, the ultrasonic power W dissipated into the water was calculated as [34]:

(16) $$W=\frac{\Delta T}{\Delta t}mC_{p},$$

where ΔT/Δt is the temporal rate of the temperature (T) rise, m is the mass of water, and $C_{p}=$ 4.2 J/g K is the specific heat of water at constant pressure. The initial temperature rise was measured at room temperature by using a DS18B20 digital thermometer, which was immersed in the water and was held at its half height. The resulting acoustic power was set to about 20 W, which corresponded to acoustic power densities of ≈1 W/mL at both the frequencies used.

However, this value gives the net ultrasonic power dissipated in the liquid [35]. So, another test of this type, estimating the sonochemical effectiveness, was made using a KI oxidation dosimetry technique. The iodide ions (*I*^−^) in the aqueous solution of KI can be transformed into iodine molecules (*I*_2_) under sonication. If excess I^−^ ions are present in the solution, *I*_2_ will further react with them thus forming the triiodide ions I_3_^−^ as [36]

(17) $$I_{2}+I^{-}↔I_{3}^{-}$$

The formation of the yellow complex *I*_3_^−^ in the colored solution was controlled by the characteristic optical absorption peaks at about 290 and 350 nm [37,38]. In these experiments, concentration of KI in water was 0.1 mol/L. Exposure to ultrasound changed initially clear solution to yellow colored. The absorption spectra were measured using an Evolution 600 Spectrophotometer. The spectral changes were found to be quite similar for sonication at 28 and 400 kHz but the peak absorption in the 350 nm band was as much as several times greater at 400 kHz than that at 28 kHz.

Within the frame of fluid mechanics, cavitation can be described as being due to the impulsive formation of cavities caused by tensile forces in high-speed flows or flow gradients arisen in a liquid. The flow pattern can be related to the characteristic distribution of nodes and antinodes along the length of the oscillating liquid. Consequently, the distribution of sound pressure at the transducer fundamental frequency plays an important role for various sonochemical reactions [39,40]. The measured sound pressure is quite accurate and comparable to that simulated numerically [40,41]. However, spatial areas of high pressures do not necessarily correspond to the ones with high reaction rates because the reaction is mediated by the bubbles. In the ultrasound process, various microbubbles inside of solvent vapor are formed, and that produces acoustic energy with radial motion through the reaction medium. Moreover, various microbubbles, from 4 to 300 μm in diameter, can be formed inside the solvent vapor, thus producing acoustic energy flows in the radial direction [42,43]. When the resonance frequency of the microbubbles exceeds that of the ultrasonic field, they collapse, which triggers the biochemical or thermochemical reactions [22]. We did not address this issue here, restricting ourselves to describing the pressure distributions at the transducer resonance frequencies.

**Figure 4:**
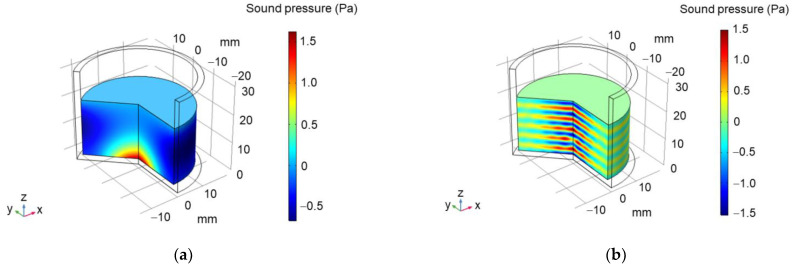
Simulated distribution of sound pressure at the driving frequency of 28 (**a**) and 400 kHz (**b**). The flask is 20 mm in diameter, 32 mm in height, with the wall thickness of 2 mm. The numerical simulations were performed using the COMSOL software.

The results of our numerical simulations are shown in Figure 4. Following extensive discussions, one may expect that the bubbles are repelled from the pressure antinodes due to Bjerknes force and settled at locations between the pressure antinodes and nodes [40]. In our experiments, the wafer sample was placed just in the region between the antinodes and nodes when using the lower frequency sonication. At about 400 kHz, it resided near the bottom of the flask shown in Figure 4.

### 2.2. Surface Processing and Photovoltage Measuring

**Figure 5:**
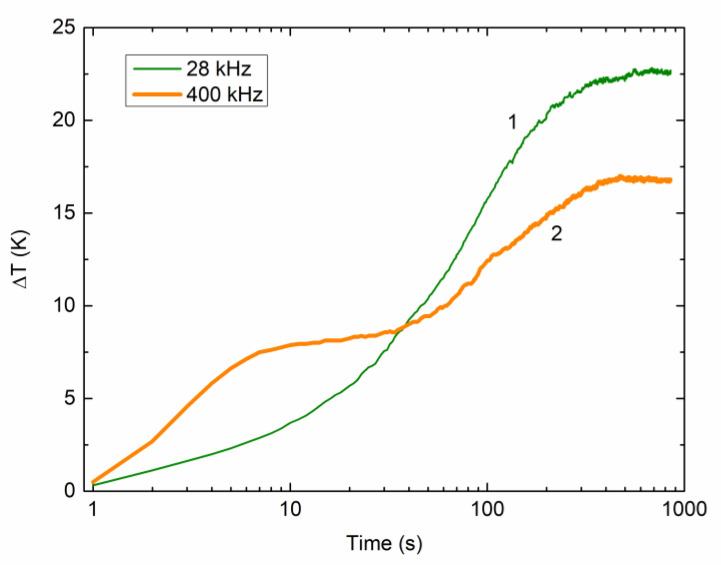
Variation of solution temperature with time after the sonication at 28 (curve 1) and 400 (2) kHz is turned on.

The measurements were performed on p-type, 1–20 Ω cm, Cz-Si(111) wafers with a thickness of 330 μm. Distilled water was used as a solvent. All reagents were analytical grade and used as purchased without further purification and treatment. All the weight measurements were done at about 20 °C using an analytical balance. The freshly prepared mixtures were stirred for about 1 min and then ultra-sonicated for 15 min to ensure solvent homogeneity. Reactant solutions were not intentionally air-saturated before their use and used within 30 min after the preparation. The sample temperature was kept constant at about 310 K using a fan heatsink. The DS18B20 temperature probe was placed in the irradiated solution to control the temperature inside it. The temperature monitoring curves for the solution irradiated at 28 and 400 kHz are shown in Figure 5. Before the measurements, the temperature of the liquid inside the flask was the ambient temperature. After the sonication was turned on, the temperature increase ΔT was monitored, as shown by curves 1 and 2 in Figure 5. At both frequencies, the solution temperature was stabilized at the larger value of ΔT≈ 17–22 K about 6 min after starting the sonication. After the temperature stabilization, the sample was placed into the solution, and the wafer surface processing was performed.

Before the chemical and sonochemical processing, the Si wafers were cleaned by a cleaning procedure, which can include rinsing with water, methanol, acetone, methanol, and finally water [44,45]. Here, we used tetrahydrofuran instead of acetone. Following the above cleaning treatments, sonication was performed in tetrahydrofuran (THF) C_4_H_8_O, methanol CH_3_OH and in a solvent mixture containing THF and methanol (THF/methanol volume ratio was equal to 3/1 and 1/1) for 15 min.

The surface photovoltage transients were measured in the contactless capacitor arrangement using the pulsed modulated light and a 100-μm glass dielectric spacer as described elsewhere [46]. The decay of the SPV was taken after the light emitting diode (center wavelength at 405 nm) was switched off.

## 3. Results and Discussion

Illumination of the silicon surface induces excess free carriers, which, being separated in the near-surface region, cause an occurrence of the surface photovoltage. The surface-sensitive SPV can therefore be used to investigate the influence of important wet-chemical treatments on the electronic surface properties as well as the hydrogen and oxide coverage [47].

The surface passivation implies the achievement of a rather long effective minority carrier lifetime $\tau_{e}$ ranging on a time scale of milliseconds, which is affected by the contribution from both the surfaces and the bulk $\tau_{b}$ as [48]:

(18) $$\frac{1}{\tau_{e}}=\frac{1}{\tau_{b}}+\frac{2\upsilon_{eff}}{W},$$

where $\upsilon_{eff}$ is the effective surface recombination velocity and W is the wafer width. It is therefore clear that the surface recombination processes, which are accounted for in Equation (18) by introducing $\upsilon_{eff}$, point to a fundamental physical limitation in increasing $\tau_{e}$. The problem can be circumvented by employing surface processing, e.g., sonication, which affects the carrier recombination velocities at the surface.

**Figure 6:**
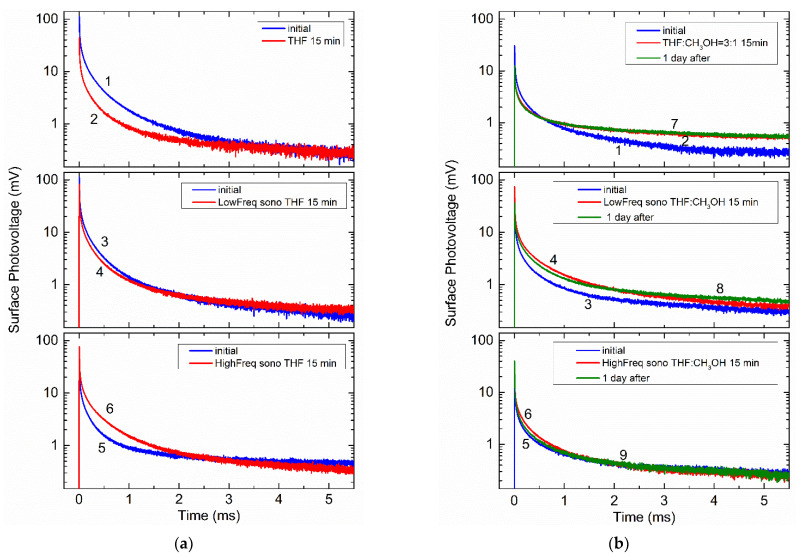
Time-dependent SPV of Si samples before (curves 1, 3, and 5) and after chemical (curves 2) and sonochemical treatments at about 28 kHz (curves 4) and 400 kHz (curves 6): (**a**) in tetrahydrofuran; (**b**) in tetrahydrofuran/methan ol mixture with a volume ratio of 3/1. Green curves 7–9 exemplify the SPV evolution one day after appropriate chemical and sonochemical treatments were made.

Figure 6a shows the variation of the SPV decays taken before (curves 1, 3, and 5) and after its chemical (curve 2) and sonochemical (curves 4 and 6) treatments in tetrahydrofuran. Exposure to lower-frequency sonication at about 28 kHz yields decay 4, while higher-frequency treatments at about 400 kHz result in decay 6 in Figure 6a. Figure 6b displays appropriate results obtained in a mixture of tetrahydrofuran and methanol. In order to check the post-treated evolution of the decays, the SPV transients were collected one day after appropriate chemical and sonochemical treatments had been made. Typical results obtained for one sample set are shown by curves 7–9 in Figure 6b, illustrating that allowing the treated sample to stay at rest was not able to remove the procession-induced changes sufficiently.

**Figure 7:**
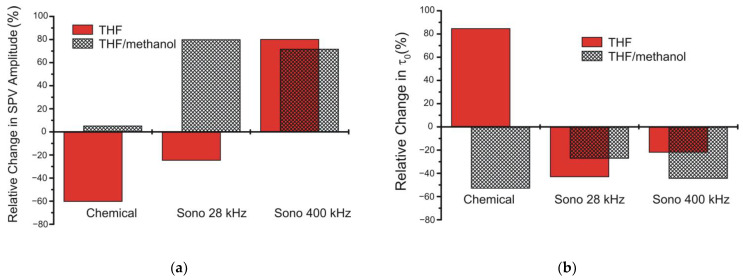
Relative change in the SPV amplitudes (**a**) and decay times $\tau_{0}$ (**b**) due to exposure of Si to tetrahydrofuran and sonication in tetrahydrofuran at about 28 and 400 kHz (red rectangles). Black rectangles are measured when using a mixture of tetrahydrofuran and methanol. The data in (**a**) are obtained from Figure 4 at time moment t= 0 and that in (**b**) by fitting the decays in Figure 6a to Equation (19).

The most prominent effect in Figure 6a is the remarkable SPV enhancement due to increasing sonication frequency. More conveniently, this can be seen in Figure 7a, showing the relative change in the SPV amplitudes $U_{0}$ taken at time t= 0, just when the light is turned off, due to chemical treatment and sonication in THF. It is seen that chemical treatment in tetrahydrofuran quenches the SPV signal (at most a 60% decrease in Figure 7a). A noticeably smaller decrease, approximately 27%, is achieved sonochemically with a frequency of 28 kHz. In marked contrast, the SPV response is increased up to ≈80% due to sonication at 400 kHz.

The time-dependent SPV decays U(t) can be written as a product of the amplitude value of the SPV signal $U_{0}$ and the shapefunction η (t), $U\left(t\right)=U_{0}\eta \;\left(t\right)$. Depending on the separation and recombination processes associated with photoexcited electrons and holes, η(t) can be approximated, e.g., by the single- or multiexponential form.

Following the analysis given elsewhere [49,50], a stretched-exponent decay model was used here, so that the SPV signal U(t) is described by:

(19) $$U\left(t\right)=U_{0}e^{-{\left(t/\tau_{0}\right)}^{\beta }},$$

where $\tau_{0}$ is the characteristic stretched-exponent decay time and β is the dispersion factor, which describes the spread of time constants. Obviously, β= 1 for a monoexponential decay, whereas the values 0<β<1 describe a variation from a monoexponential decay with smaller β values corresponding to a broader distribution of decay times. All the fitting results are summarized in Table 1.

It is seen in Table 1 that the factor β is not affected greatly under different treatments in tetrahydrofuran and is approximately equal to 0.3 for the data shown in Figure 6a. In contrast, the decay time $\tau_{0}$ varies rather significantly upon different kinds of the treatments. After chemical treatment in tetrahydrofuran, the time constant sharply increases by about 80% from its initial value. In turn, the sonication in THF leads to shortening of the decay, more pronounced for the sonochemical processing at about 28 kHz.

The likely mechanism that has come to describe the previous observations with chloroform CHCl_3_ and dichloromethane CH_2_Cl_2_ relies on the fact that these solutions act as sources of carbon, which can saturate the dangling bonds revealed on the surface of Si, thus passivating the surface [15,51]. In this scenario, using CHCl_3_ or CH_2_Cl_2_ yields the Si–H and C–Cl bonds, which react, forming C–H species. This, in turn, resembles the chlorination/alkylation process that forms Si−alkyl, converting Si–H into Si–C*_n_*H_2*n*+1_ (n≥1). The alkyl chains on Si surfaces are known to provide low surface recombination velocities [52], thus featuring effective Si surface passivation [44].

Too little is still known about the nature and mechanisms of formation of the initial radicals and molecules produced at thermal decomposition of the tetrahydrofuran ring [53,54,55]. Meanwhile, the overall pyrolysis of THF can be summarized by the following major participating reactions [54]:

(20) $$\mathrm{THF}\to {\mathrm{CH}}_{2}={\mathrm{CH}}_{2}+{\left({\mathrm{CH}}_{2}\right)}_{2}-O,$$

(21) $${\left({\mathrm{CH}}_{2}\right)}_{2}-O\to \mathrm{HCO}+{\mathrm{CH}}_{3},$$

(22) HCO→H+CO,

(23) $$\mathrm{THF}\to {\mathrm{CH}}_{3}-\mathrm{CH}={\mathrm{CH}}_{2}+{\mathrm{CH}}_{2}O,$$

(24) $${\mathrm{CH}}_{3}-\mathrm{CH}={\mathrm{CH}}_{2}\to {\mathrm{CH}}_{2}=C={\mathrm{CH}}_{2}\to {\mathrm{CH}}_{3}-C\equiv \mathrm{CH},$$

(25) $${\mathrm{CH}}_{3}-\mathrm{CH}={\mathrm{CH}}_{2}+{\mathrm{CH}}_{3}\to {\mathrm{CH}}_{3}-{\mathrm{CH}}_{2}-\mathrm{CH}={\mathrm{CH}}_{2}+H$$

Here, Equation (24) gives a schematic reaction presentation. It is seen in Equation (22), Equation (24), and Equation (25) that hydrogen and carbon (as well as carbon monoxide) can be among the by-products. Therefore, the atomic hydrogen is able to effectively passivate dangling bonds at the Si surface, suppressing the surface state density and surface recombination velocities [56], and hence enhancing the SPV response. Furthermore, carbon atoms at the surface create Si−C bonds and dangling carbon bonds, which are then saturated by H atoms.

**Figure 8:**
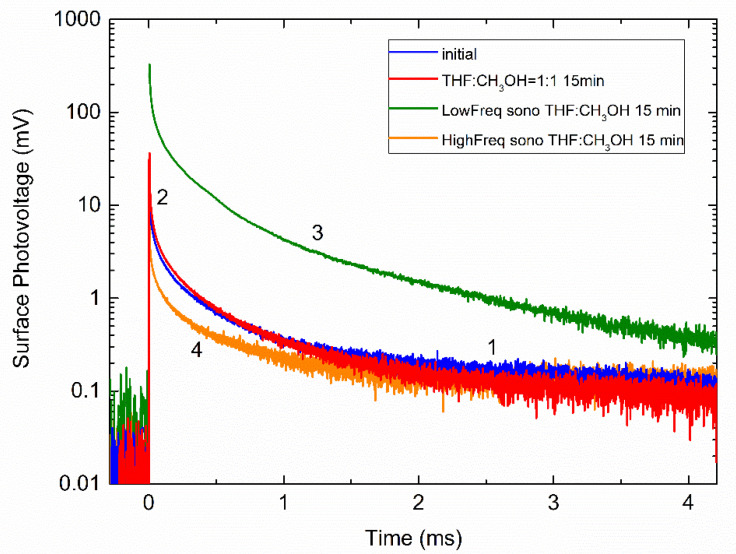
Time-dependent SPV of Si samples before (curve 1) and after chemical (curve 2) and sonochemical treatments at about 28 (curve 3) and 400 kHz (curve 4) in tetrahydrofuran/methanol mixture with a volume ratio of 1/1.

Methanol can be used to dilute the solvent and adjust the molar concentration of active radicals decomposed from tetrahydrofuran. Figure 8 and the black rectangles in Figure 7 illustrate how sensitive is the ultrasonic effect to the ratio of THF/methanol and shows that the chemical treatment of the Si surface has an opposite effect in THF and THF/methanol. The decay time $\tau_{0}$ in a mixture of tetrahydrofuran and methanol given in Table 1 behaves quite similarly under the chemical and sonochemical treatments. Therefore, slight variation of the THF concentration does not change appreciably the relative weights of the decay components, slightly varying the values of $\tau_{0}$ that might be related to a solvent polarity [57]. Increasing the volume of methanol in the mixture to the ratio of 1/1 gives the changes in the SPV amplitudes (see Figure 8), which are rather similar to that observed in the 3/1 mixture (Figure 7a) in cases of the chemical treatment (curve 2 in Figure 8) and sonication at 28 kHz (curve 3). The higher-frequency treatment quenches the SPV signal, as shown by curve 4 in Figure 8. In contrast, the decay time $\tau_{0}$ exhibits a decrease when treating chemically (from 33.7 to 13.4 μs) or sonochemically at 28 kHz (from 26.2 to 17.8 μs), while $\tau_{0}$ is enhanced by increasing the sonication frequency (from 19.2 to 24.9 μs in our measurements).

**Figure 9:**
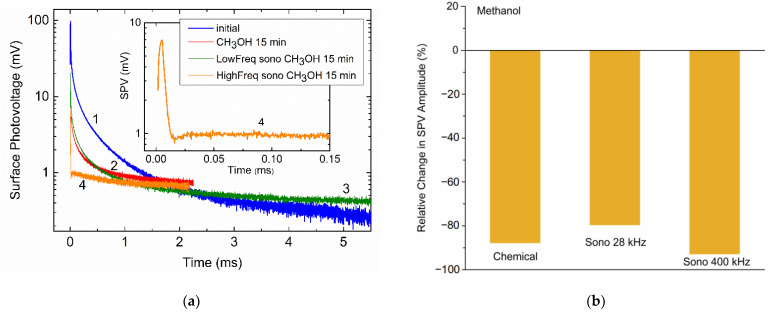
(**a**) Time-dependent SPV of Si samples before (curve 1) and after chemical (curve 2) and sonochemical treatments at about 28 (curve 3) and 400 kHz (curve 4) in methanol; (**b**) relative change in the SPV amplitudes due to exposure of Si to methanol and sonication in methanol at about 28 and 400 kHz. The data in (**b**) are obtained from decays shown in (**a**) at time moment t=0.

Most significantly, the SPV amplitude is similarly enhanced in the 3/1 mixture upon sonication at both 28 and 400 kHz, as shown by black rectangles in Figure 7a. Furthermore, the SPV amplitude is effectively quenched both chemically and sonochemically in pure methanol, as shown in Figure 9a,b.

The essence of these observations lies in the fact that methanol itself can probably be decomposed [58] ultrasonically:

(26) $${\mathrm{CH}}_{3}\mathrm{OH}\to 2H_{2}+\mathrm{CO}$$

The difference between sonication in tetrahydrofuran and methanol might therefore be caused by the occurrence of the atomic hydrogen released from THF in the reaction given by Equation (22), which provides an effective passivation of the Si surface. Based on the fact that the H-bonded methanol dimer is formed [59,60] and succeeded in decreasing passivation ability of hydrogen, a slight decrease in the SPV signal in THF/methanol observed in Figure 7a at Sono 400 kHz can naturally be explained.

The interesting feature is the ultrasonic frequency effect observed in Figure 7a for the red and black rectangles. Such an effect might be driven by the complex dissociation dynamics in THF and THF/methanol mixtures [61,62,63]. It may be suggested that some kind decay process with hydrogen happens in the time window between 36 μs (corresponding to 28 kHz) and 2.5 μs (400 kHz).

Another major effect of sonication in methanol, which is most clearly seen in curve 4 of Figure 9a (also enlarged in the inset) taken at about 400 kHz, is a complicated shape of the decay. This seemingly stems from the fact that this SPV decay can be decomposed into two transients with positive and negative values of the partial amplitude factors $U_{01}$ and $U_{02}$. This suggests that, upon the higher-frequency sonication in methanol, there is a competitive path of separating photoexcited electrons and holes on the surface, which is heavily blocked by lowering the sonication frequency. The origin of this behavior is not precisely determined and a more detailed examination of it, correlating the decay shape transformation with the sonication frequency and testing whether these data are mutually consistent, is warranted.

## 4. Conclusions

Here, we sonicated tetrahydrofuran, which can act as a source of hydrogen capable of improving the photovoltaic response of Si wafers. Two ultrasonic frequencies of about 28 and 400 kHz were employed. The enhanced SPV signal was observed due to increasing sonication frequency. Thus, the SPV response was increased up to ≈80% due to sonication at 400 kHz, whereas the signal decreased down to ≈75% of the initial value when the frequency was lowered to about 28 kHz. The addition of methanol was found to suppress the significant difference in the sonication effect at the two frequencies.

Notwithstanding the many scenarios that might be involved in properly interpreting the observed frequency behavior, this work hinted at the possibility of involvement of atomic hydrogen decomposed from tetrahydrofuran. This is known as effective passivator of surface silicon dangling bonds. It is therefore suggested that different decay processes with hydrogen occur over a physically meaningful time scale from 2.5 to 36 μs, which corresponds to the ultrasonic frequencies of 400 and 28 kHz, respectively.

## Figures and Tables

**Table 1:** The peak amplitudes $U_{0}$ of the SPV signals and fitting parameters $\tau_{0}$ and b for the decays shown in Figure 6.

Treatment	Peak Amplitude (mV)	$\tau_{0}\;(\mu s)$	β
THF Figure 4a	112.8 (curve 1)	6.0	0.294
	44.4 (curve 2)	11.1	0.329
	111.5 (curve 3)	9.0	0.321
	83.6 (curve 4)	5.1	0.288
	41.9 (curve 5)	10.8	0.335
	75.6 (curve 6)	8.4	0.296
THF/Methanol (3/1) Figure 4b	14.6 (curve 1)	6.4	0.302
	15.4 (curve 2)	3.0	0.247
	41.4 (curve 3)	7.3	0.307
	74.6 (curve 4)	5.3	0.275
	20.0 (curve 5)	10.3	0.309
	34.4 (curve 6)	5.7	0.274

## Data Availability

The data presented in this study are available on request from the corresponding author.

## Sample Availability

Samples of the compounds are not available from the authors.
